# Dr. James H. Hewitt

**Published:** 1912-04

**Authors:** 


Dr. James H. Hewitt, a classmate of Dr. Lapp's died at his home in Lebanon, Ill., February 25, 1912, aged 71. He was Surgeon in the 147th Volunteers of Pennsylvania, during the Civil War and later practiced medicine in Summerfield, Ill., for more than 40 years.

